# The efficacy of adipose-derived stem cells in burn injuries: a systematic review

**DOI:** 10.1186/s11658-023-00526-w

**Published:** 2024-01-05

**Authors:** Michael Kohlhauser, Alexandru Tuca, Lars-Peter Kamolz

**Affiliations:** 1 Division of Plastic, Aesthetic and Reconstructive Surgery, Department of Surgery, Medical University of Graz, Graz, Austria; 2 Department of Surgery, State Hospital Güssing, Güssing, Austria; 3 COREMED-Cooperative Centre for Regenerative Medicine, JOANNEUM RESEARCH Forschungsgesellschaft mbH, Graz, Austria

**Keywords:** Adipose-derived stem cells, Mesenchymal stem cells, Stem cell research, Burns, Burn injury, Burn care, Tissue engineering, Regenerative medicine, Wound healing

## Abstract

**Background:**

Burn injuries can be associated with prolonged healing, infection, a substantial inflammatory response, extensive scarring, and eventually death. In recent decades, both the mortality rates and long-term survival of severe burn victims have improved significantly, and burn care research has increasingly focused on a better quality of life post-trauma. However, delayed healing, infection, pain and extensive scar formation remain a major challenge in the treatment of burns. ADSCs, a distinct type of mesenchymal stem cells, have been shown to improve the healing process. The aim of this review is to evaluate the efficacy of ADSCs in the treatment of burn injuries.

**Methods:**

A systematic review of the literature was conducted using the electronic databases PubMed, Web of Science and Embase. The basic research question was formulated with the PICO framework, whereby the usage of ADSCs in the treatment of burns in vivo was determined as the fundamental inclusion criterion. Additionally, pertinent journals focusing on burns and their treatment were screened manually for eligible studies. The review was registered in PROSPERO and reported according to the PRISMA statement.

**Results:**

Of the 599 publications screened, 21 were considered relevant to the key question and were included in the present review. The included studies were almost all conducted on rodents, with one exception, where pigs were investigated. 13 of the studies examined the treatment of full-thickness and eight of deep partial-thickness burn injuries. 57,1 percent of the relevant studies have demonstrated that ADSCs exhibit immunomodulatory effects during the inflammatory response. 16 studies have shown improved neovascularisation with the use of ADSCs. 14 studies report positive influences of ADSCs on granulation tissue formation, while 11 studies highlight their efficacy in promoting re-epithelialisation. 11 trials demonstrated an improvement in outcomes during the remodelling phase.

**Conclusion:**

In conclusion, it appears that adipose-derived stem cells demonstrate remarkable efficacy in the field of regenerative medicine. However, the usage of ADSCs in the treatment of burns is still at an early experimental stage, and further investigations are required in order to examine the potential usage of ADSCs in future clinical burn care.

## Introduction

Burn injuries are unpredictable traumas by their nature, and have varying degrees of severity. As with all wounds, the healing process of burns involves dynamic and overlapping phases including inflammation, proliferation and remodelling [1]. While partial thickness wounds can heal within 14 days with less scarring, deep partial and full-thickness burns are associated with prolonged healing, infection, an extensive inflammatory response and pathological scarring [1, 2]. Over the past decades, good progress has been made in the acute treatment of burn injuries. The mortality rate as well as the long-term survival of severely burned patients have improved significantly [3]. In recent years, burn care research has shifted to a better quality of survival by focusing on improvement wound healing, scar quality and contracture prevention [4]. However, delayed healing, infection, pain and pathological scar formation remain major challenges in burn care [1, 2]. The ultimate goal is to develop novel therapies that support the healing process and enable improved treatment outcomes.

Mesenchymal stem cells (MSCs) have emerged as a novel therapeutic approach in wound care and tissue regeneration [5, 6]. A distinct type of MSCs was discovered in large quantities within adipose tissue, namely adipose-derived stem cells (ADSCs) [7, 8].The effectiveness of ADSCs application in wound healing, including an improved immunoregulation, neovascularisation, granulation tissue formation, re-epithelialisation and remodelling, as well as their differentiation potential in various cell types was proven in several in vitro and in vivo studies [9–15]. The aim of this review is to evaluate the efficacy of adipose-derived stem cells in the treatment of burn injuries.

## Methods

The present systematic review was registered in the PROSPERO database (CRD42022364221) and conducted following a protocol guided by the Preferred Reporting Items for Systematic Reviews and Meta-Analyses (PRISMA) Statement [16].

### Identify the research question

The fundamental research question was formulated with the PICO framework as follows: How effective are adipose-derived stem cells in the treatment of burn injuries in vivo? The creation process is illustrated in Table 1.

**Table 1:** Creation of the research request according to the PICO framework

Population	Intervention	Control	Outcome
In vivo wound models	ADSCs application	Control group	improved wound healing
How effective are adipose-derived stem cells in the treatment of burn injuries in vivo?			

### Search strategy

A systematic review of the literature was performed, in order to detect concerns from in vivo studies published up to 30th September 2022 on the efficacy of adipose-derived stem cells in the treatment of burn injuries. To identify appropriate studies, the following online databases were searched: PubMed, Web of Science and Embase. The key terms of the applied search strategy for each online databank are displayed in Table 2. Additionally, pertinent journals that focus on burn care research were searched manually.

**Table 2:** Key terms of the applied search strategy

Key terms	
PubMed	("adipose-derived stem cells" OR "adipose tissue derived stem cells" OR "adipose-derived mesenchymal stem cells" OR "adipose derived mesenchymal stem cells" OR "adipose tissue-derived mesenchymal stem cells" OR "adipose tissue derived mesenchymal stem cells" OR "adipose stem cells" OR "adipose mesenchymal stem cells") AND ("burns" OR "burn injury" OR "burn injuries" OR "thermal injury" OR "thermal injuries")
Web of Science	("adipose-derived stem cells" OR "adipose-derived mesenchymal stem cells" OR “adipose tissue stem cells” OR “adipose mesenchymal stem cells”) AND ("burns" OR "burn injury" OR "burn injuries" OR "thermal injury" OR "thermal injuries")
Embase	((adipose-derived stem cells or adipose derived stem cells or adipose mesenchymal stem cells or adipose tissue stem cells) and burns).af

### Study selection

The usage of adipose-derived stem cells in the treatment of burns was determined as the fundamental inclusion criteria. Firstly, all search results were exported into Mendeley Desktop (Version 1.19.8) and duplicates were eliminated. In the next step, titles, abstracts, and later, full-text articles, were analysed in relation to the inclusion criteria. Only full-text original articles published in the English language were eligible. Case reports, review articles, letter and comments, but also non in vivo studies were excluded. Furthermore, publications in which outcomes of ADSC therapy had inadequate focus or were not declared as primarily responsible for the treatment outcome were excluded. To ensure no inequity by wrongful exclusion, the whole analysis was performed by two investigators. In the event of a consensus between the two researchers being was found, a publication was included in the review process. With regard to missing or unclear information, the corresponding authors were contacted once by-email.

Study inclusion criteria:

- Randomized and non-randomized controlled in-vivo studies
- Only full-text original articles published in the English language will be eligible
- Studies must focus on adipose-derived stem cells in the therapy of burns in vivo.

Study exclusion criteria:

- Clinical trials
- Ex vivo studies
- Case reports
- Reviews
- Letters and comments
- Paper not available as full-text
- Paper not published in English language
- Wounds not classified as burns
- Depth extent of the injury is less than deep partial
- The exact depth of the wound is not specified
- ADSCs were not declared as primarily responsible for the treatment outcome

### Data extraction

The following data were extracted from the text, tables, and graphs of the eligible studies by two independent study associates: (1) Study design; (2) Animal model; (3) Conditions of the wound; (4) Origin of ADSCs; (5) Dosage of ADSCs; (6) Carrier medium; (7) Method of application; (8) Comparison group; (9) Study duration; (10) Measured outcomes. Table 3 encompasses the key data extracted from the included studies.

**Table 3:** Characteristics of studies included

Study	Year	Animal model	Wound conditions	ADSCs origin	ADSCs-Dose	Carrier	Application	Comparison group	Duration	Outcome
Oryan et al. [17]	2019	Animal: Sprague–Dawley rats Quantity: 12 Age: 7–9 weeks Weight: 180–220 g Gender: male	Four circular full thickness burn wounds 10 mm in diameter were created on the back of each rat by an aluminium bar (100 °C) Prior to the treatment, a debridement was performed	Allogeneic Rat ADSCs	1 × 10^6^ cells	Aloe vera hydrogel	Intradermal injection	• DBM-Aloe vera/ADSCs • DBM-Aloe vera • DBM • Aloe vera	7, 14 and 28 days	• Accelerated wound closure • reduced Inflammatory cells • IL-1β ↓ • TGF-β1 ↑ • bFGF↑ • Increased neovascularisation • more organised collagen • fibroblasts ↑ • Increased re-epithelialisation
Zhou et al. [18]	2019	Animal: Sprague Dawley rats Quantity: 27 Age: 1 year Weight: No information given Gender: male	A third-degree burn wound (2cm^2^) was created by putting the head of a temperature-controlled desktop scalding instrument (100 °C) on the back of each rat with a pressure of 1000 g	Autologous Rat ADSCs	2 × 10^6^ cells	Resuspended in 500 μl PBS	Subcutaneous injection	• ADSCs single injection • ADSCs multiple injection • Control	3, 12, 21 and 27 days	• Accelerated wound closure • Increased neovascularisation • VEGF ↑ • IL-1ra ↑
Oryan et al. [19]	2019	Animal: Sprague–Dawley rats Quantity: 12 Age: 7–9 weeks Weight: 180–220 g Gender: male	Four circular full thickness burn wounds 10 mm in diameter were created on the back of each rat by an aluminium bar (100 °C) Necrosis was removed 48 h post-burn	Allogeneic Rat ADSCs	1 × 10^6^ cells	Tegaderm-honey	Intradermal injection	• Tegaderm-honey-ADSCs • Tegaderm + honey • Tegaderm • Honey	7, 14, and 28 days	• Accelerated wound closure • wound closure rate ↑ • Reduced Inflammatory cells • bFGF ↑ • TGF-β1 ↑ • IL-1β ↓ • blood vessels ↑ • Increased granulation tissue formation • Increased re-epithelialisation • More organised collagen • Hair follicle ↑
Kaita et al. [20]	2019	Animal: BALB/c nude mice Quantity: 18 Age: 6–8 weeks Weight: Information is not given Gender: male	Full-thickness burn wounds were created by placing a pre-heated (150 °C) aluminum column (6 mm in diameter) on the dorsum for 5 s	Human ADSCs	5 × 10^4^ cells	Terudermis™ Artificial dermis	Direct application of ADSCs loaded artificial dermis	• Fresh ADSCs • Frozen ADSCs • control	• 6 and 12 days	• Reduced wound surface • Increased skin thickness • Increased neovascularisation • HGF ↑ • Increased collagen synthesis
Banerjee [21]	2019	Animal: Harlan rats Quantity: 16 Age: 8–10 weeks Weight: Information is not given Gender: male	A circular deep partial thickness burn was created using a brass soldering device (17 mm diameter) heated to 87 °C. The device was applied to the dorsal area of rats for 10 s with a constant force of 500 g using a weighted soldering tip. After 12 h, the wounds of groups 2–4 were infected with P. aeruginosa	Rat ADSCs “no mention of whether allogeneic or autologous source”	5 × 10^4^ cells	PEGylated fibrin hydrogel containing of silver sulfadiazine (SSD) loaded chitosan microsphere	Application within Hydrogel as wound dressing	• Uninfected control • Infected saline • Infected PEGylated fibrin hydrogel containing of silver sulfadiazine (SSD) loaded chitosan microsphere • Infected PEGylated fibrin hydrogel containing of silver sulfadiazine (SSD) loaded chitosan microsphere with ADSCs	1, 4, 7, 14, 21 and 28 days	• Increased neovascularisation • Increased collagen deposition • More organized & thicker granulation tissue • Increased re-epithelialisation
Loder et al. [22]	2014	Animal: C57BL/6 mice Quantity: 20 Age: 6–8 weeks Weight: 20-25 g* Gender: male	All animals underwent a 30% surface area partial- thickness scald injury by exposure to 60 °C water for 17 s	Allogeneic Mouse ADSCs	1 × 10^6^ cells	Suspended in 500 μl PBS	Subcutaneous injection	• Processed adipose tissue • ADSCs • Adipose tissue/ADSCs • sham	5 and 14 days	• Decreased wound depth on day 5 • Decrease in cell apoptosis • Increased neovascularisation
Motamed et al. [23]	2017	Animal: Sprague–Dawley rats Quantity: 32 Age: Information is not given Weight: 280–320 g Gender: male	A full thickness burn injury was induced on each rat by laying a bar with four columns (10 × 20 mm), which was kept in boiling water for 5 min on the dorsum for 30 s	Human ADSCs	5 × 10^5^ cells	Human amniotic membrane	Direct application of ADSCs loaded human amniotic membrane	• Vaseline gauze (control) • human amniotic membrane (HAM) • fetal fibroblasts seeded on HAM • ADSCs seeded on HAM	0, 7, 14, 20, 28, 40, 50 and 60 days	• Accelerated re-epithelialisation • reduced inflammatory cell • less fibrosis • decreased healing time
Gholipourmalekabadi et al. [24]	2018	Animal: BALB/c male mice Quantity: 75 Age: 4–6 weeks* Weight: 16–19 g* Gender: male	Two full-thickness burns (1 cm in diameter) were produced on the back of each mouse by placing a heated metal sheet (100 °C) for 10 s The necrotic tissue resulting was meticulously debrided before fat grafting	Allogeneic Mouse ADSCs	1 × 10^4^ cells	Bi-layered 3D artificial skin made from decellularized human amniotic membrane	Direct application of ADSCs loaded artificial skin	• Control • human amniotic membrane (AM) • ADSCs/AM • AM/ESF • ADSCs/AM/ESF	7, 14 and 28 days	• Accelerated wound closure • Support early inflammatory response • Increased neovascularisation • Increased re-epithelialisation • Reduced scar elevation index (SEI) • Hair follicle ↑ • TGF-β1 ↑ • MIP2 ↓ • TNF-α1 ↓ • MMP-1 ↑ • MMP-2 ↑ • IL-1β ↑ • bFGF ↑
Bliley et al. [25]	2016	Animal: athymic nude mice Quantity: 24 Age: 7–9 weeks Weight: 25-30 g* Gender: female	A full-thickness burn (1 cm diameter) injury was created on the dorsum of each mouse by a brass stamps, which were wrapped in aluminium foil and heated overnight in a Fisher convection oven at 70 °C	Human ADSCs	6.8 × 10^6^ cells	Resuspended in o.5 ml PBS	Subeschar injection	• Control (PBS) • ADSCs/PBS	4, 7, 14, and 21 days	• Increased collagen formation • Increased neovascularisation • Collagen I ↑ • Collagen III ↑ • Higher ratio of III to I collagen
Alemzadeh et al. [26]	2019	Animal: Sprague–Dawley male rats Quantity: 12 Age: 8 weeks Weight: 200–250 g Gender: male	Three circular full-thickness burn injuries (10 mm in diameter) were created on the back of each rat by an aluminum bar boiled in 100 °C water for 30 s Necrosis was removed 48 h post-burn	Allogeneic Rat ADSCs	1 × 10^6^ cells	Hyaluronic acid hydrogel	Intradermal injection and topical application	• ADM • Hyaluronic acid + ADM • Hyaluronic acid/ADSCs + ADM	0, 7, 14, and 28 days	• Accelerated wound closure • IL-1β ↓ • TGF-β1 ↑ • bFGF ↑ • Fibroblasts ↑ • Increased neovascularisation • Increased re-epithelialisation • Accelerated remodelling
Dong et al. [27]	2020	Animal: FVB/NJ mice Quantity: 15 Age: 10–12 weeks Weight: Information is not given Gender: female	Two deep second-degree burns were made on the dorsum of each mouse by using an aluminium cylinder (10 mm diameter) heated in a 100 °C water bath for 10 min Two days after burning, all necrotic tissue was debrided to create a fresh full-thickness wound	Human ADSCs & Allogeneic Mouse ADSCs	3 × 10^5^ cells	Hydrogel	Application within Hydrogel as wound dressing	• Hydrogel + ADSCs • Hydrogel • Control (no treatment)	3, 9, 11, 14 and 21 days	• Accelerated wound closure • Increased neovascularisation • Higher ratio of III to I collagen • Myofibroblasts↓
Cabello-Arista et al. [28]	2022	Animal: athymic nude mice Quantity: 25 Age: 3 months Weight: 24–26 g* Gender: male	To create a third degree burn injury a 105 °C heated cooper device (2 cm^2^) was placed on the back of each mouse for 5 s and quickly removed Burned area was debrided with a scalpel to remove necrotic tissue	Human ADSCs	2 × 10^4^ cells	Radiosterilized human amnion (RHA) *or* radioster- ilized pig skin (RPS)	Direct Application with ADSCs loaded RHA or RPS	• RHA + ADSCs • RPS + ADSCs • RHA • RPS • Control (gauze with petroleum jelly)	7 and 14 days	• Improved collagen deposition • Collagen I ↑
Feng et al. [29]	2019	Animal: Sprague Dawley rats Quantity: 12 Age: Information is not given Weight: 250–300 g Gender: Information is not given	A copper plate (1 cm^2^), heated to 90ºC was applied on the dorsum of each rat for 30 s to create three deep partial thickness burns on two strips of skin islands respectively. After treatments the skin islands were embedded into the subcutaneous pockets	Rat ADSCs “no mention of whether allogeneic or autologous source”	5 × 10^5^ cells	Resuspended in 0.2 mL PBS	Intradermal injections	• ADSCs • Control	7, 14, 21 and 29 days	• Improved hair growth • Number of live hair follicle↑ • Increased neovascularisation
Barrera et al. [30]	2021	Animal: eC57BL/6 J mice Quantity: 24 Age: 8–12 weeks Weight: Information is not given Gender: Information is not given	Two partial thickness burns were created on each mouse dorsum with aluminium cylinders (10 mm in diameter) heated in a 100 °C water bath for 5 min and applied on the animals for 15 s Burns were debrided 5 days post injury using a blunt stainless steel rod	Mouse ADSCs “no mention of whether allogeneic or autologous source”	2.5 × 10^5^ cells	Hydrogel Or albumin-containing media	Application with Hydrogel as wound dressing or Injection (The location or depth of the injection was not disclosed.)	Experiment A: • ADSCs hydrogel • ASC injection • Hydrogel • Control (no treatment) Experiment B • CD26 + CD55 + ADSCs • FACS-sorted ADSCs, • unsorted ADSCs • SVF • Control (no treatment)	Experiment A: 10 and 25 days Experiment B: 29 days	• Accelerated wound closure • Increased re-epithelialisation • Increased neovascularisation • MCP-1↑ • VEGF↑ • TIMP1↓ • TNF-α↓ • Less scarring
Azam et al. [31]	2021	Animal: Wistar rats Quantity: 22 Age: 3–4 months Weight: 200–250 g Gender: male	A sterile filter paper disc (20 mm in diameter) was soaked in 12.06 N HCl (Merck, USA) for 1 min and applied at the dorsal side of the neck for 10 min to inflict the acid burn injury 24 h after injury, the necrotic tissue was carefully removed and deep partial thickness wounds were revealed	Allogeneic rat ADSCs	2 × 10^6^ cells	Dulbecco's Modified Eagle Medium (DMEM)-Low Glucose with and without 5 μM curcumin	Intradermal injection	• ADSCs • Curcumin –preconditioned ADSCs • Control (saline)	4, 8, 12, 16, 20 and 24 days	• Accelerated wound closure • Reduced inflammation cells • Increased re-epithelialisation • Increased granulation tissue formation • Increased collagen deposition • IL-1β↓ • IL-6↓ • TNF -α↓ • VEGF↑ • HGF↑ • HIF-1 –α↑ • TGF- β1↑ • FGF-2↑ • Col1α1↑
Foubert et al. [32]	2015	Animal: Gottingen minipigs Quantity: 8 Age: 5–6 months Weight: 12–16 kg Gender: female	A custom-made brass block (3.5 cm in diameter), pre-heated to 180–200 °C and weighing 350 g, was pressed onto the dorsum of the animal with a pressure of 0.4 kg/cm^2^ for 1 min to create six full-thickness burns per side were induced on the dorsum of each animal	Autologous Pig ADSCs	6,25 × 10^6^ cells	Collagen-based matrix (CBM) (i.e. Integra ®)	Direct application with the collagen-based matrix layer	• ADSCs • Control (CBM alone)	7, 14, 21 and 28 days	• Increased neovascularisation • Increased collagen deposition • More organized & thicker granulation tissue
Roshangar et al. [33]	2021	Animal: Rats Quantity: 36 Age: Information is not given Weight: Information is not given Gender: male	A full-thickness cutaneous wound of 2 × 2 cm was induced on the back of each rat through scaldingt After 24 h, the necrotic tissue was removed The temperature at which the burns were inflicted was not specified in the document	Rat ADSCs “no mention of whether allogeneic or autologous source”	1 × 10^5^ cells	3D-biorinted gel scaffold	Direct application with the scaffold	• 3D bioprinter derived‐gel scaffold + ADSC • 3D bioprinter derived‐gel scaffold • Control (no treatment)	5, 14 and 21 days	• Accelerated wound closure • Reduced inflammatory cells • Increased neovascularisation • Organized collagen deposition • Increased re-epithelialisation
Wu et al. [34]	2021	Animal: Balb/c mice Quantity: 32 Age: 8 weeks Weight: Information is not given Gender: male	After removal of the dorsal hairs, heated steel rods with a diameter of 14 mm and a temperature of 100 °C were applied for one minute to cause full thickness burns Two days post-injury, the necrotic skin was excised, and a silicone ring was stitched around the wound area for protection and containment	Allogeneic Mouse ADSCs	2 × 10^6^ cells	3D-biorinted gel scaffolds	Direct application with the scaffold	• 3D-ADSCs • 3D-NO • 3D-ADSCs/No • Control (gauze)	0, 7 and 14 days	• Accelerated wound closure • Increased neovascularisation • Increased collagen deposition • Increased re-epithelialisation
Franck et al. [35]	2019	Animal: Wistar rats Quantity: 23 Age: 3 months Weight: 250–280 g Gender: male	Burns were induced by placing a square ceramic pattern (484 mm^2^) on the abdomen, heated to 100 °C. The ceramic was applied with its own weight equivalent to 54 g for thirty seconds, resulting in a full-thickness skin injury	Allogeneic Rat ADSCs	3.2 × 10^6^ cells	DMEM	Intradermal injection	• ADSCs • Control (no treatment)	4,7 and 14 days	• Accelerated wound closure • Reduced inflammatory cells • Increased collagen deposition
Karimi et al. [36]	2014	Animal: Balb/c mice Quantity: 30 Age: Information is not given Weight: 40 g Gender: male	A heated metal probe, with a surface area of 1.5 cm^2^ and a temperature of 96 °C, was applied to the backs of the mice for 8 s to produce a standard full-thickness burn injury	Allogeneic Mouse ADSCs	1 × 10^6^ cells	Physiologic serum	Intradermal injection	• ADSCs • Adipocytes • Control (no treatment)	7, 14 and 21 days	• Accelerated wound closure • Reduce inflammatory cells • Increase re-epithelisation • Fibroblasts ↑
Ng et al. [37]	2021	Animal: C57BL/6NTac mice Quantity: 42 Age: 6–8 weeks Weight: Information is not given Gender: female	A stainless-steel bar measuring 6 mm × 5 mm and weighing 96.2 g was heated in a water bath at 100 °C for 15 min. To induce a full-thickness burn injury, the hot surface of the bar, was placed on the shaven posterior-dorsum of each mouse for a duration of 30 s The eschar was removed 48 h post-burn, directly before treatment	Human ADSCs	6 × 10^4^ cells	Hydrogel	Application with hydrogel as wound dressing	• Hydrogel/ADSCs • Hydrogel soaked in ADSCs-conditioned media • Hydrogel • Control (no treatment)	0, 7, 14 and 21 days	• Accelerated wound closure • Reduce inflammatory cells • Hair follicle & sebaceous glands ↑

*The data, not present in the publication, was procured

## Results

**Fig. 1:**
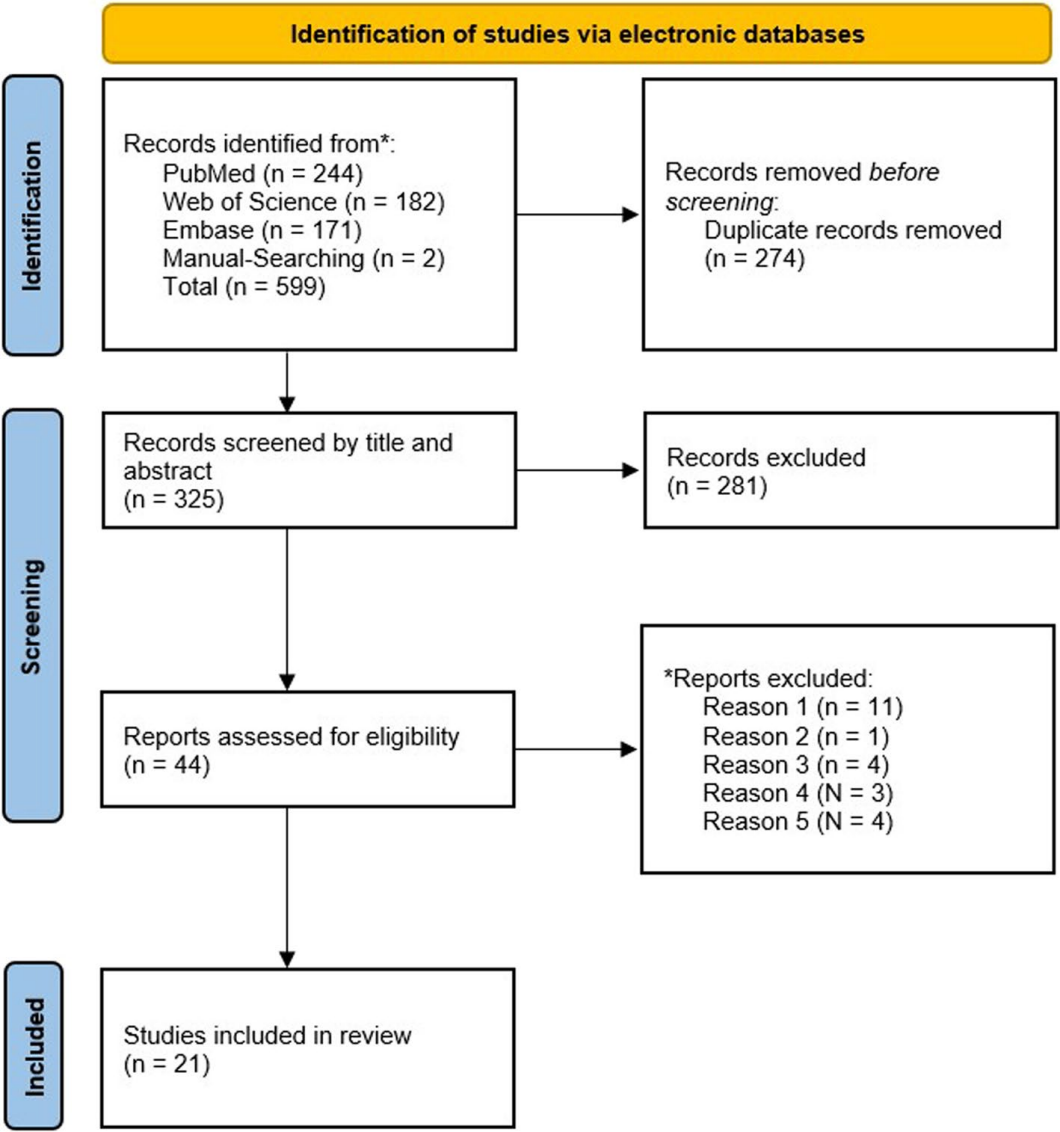
Flow diagram (Preferred Reporting Items for Systematic Reviews and Meta-Analyses—PRISMA) of the study inclusion process. *Reports excluded: Reason 1: had no or inadequate focus on inadequate focus on the efficacy of ADSCs in the treatment of burns; Reason 2: unavailable in the English language; Reason 3: irretrievable, Reason 4: no burn injury model, Reason 5: the depth extent of the burn was not defined

A total of 599 publications were identified from searches of electronic databases by using the specified search strategy. After the elimination of duplicates (n = 274), 325 publications were manually screened for relevant publications. Based on the title and the abstract, 281 were excluded due to the wrong topic or because they were not considered to be original quantitative research (e.g.; review articles, comments etc.), with 44 full text articles being retrieved and assessed for eligibility. Of these, 23 articles were excluded for the following reasons: 11 studies had inadequate focus on the efficacy of ADSCs or they were not declared as primarily responsible for the treatment outcome, one was unavailable in the English language, four were irretrievable, three had no burn injury model, and four other studies did not give clarity on wound depth. Consequently, 21 publications fulfilled the inclusion criteria. The study's inclusion process is displayed in Fig. 1.

### Bias assessment

**Fig. 2:**
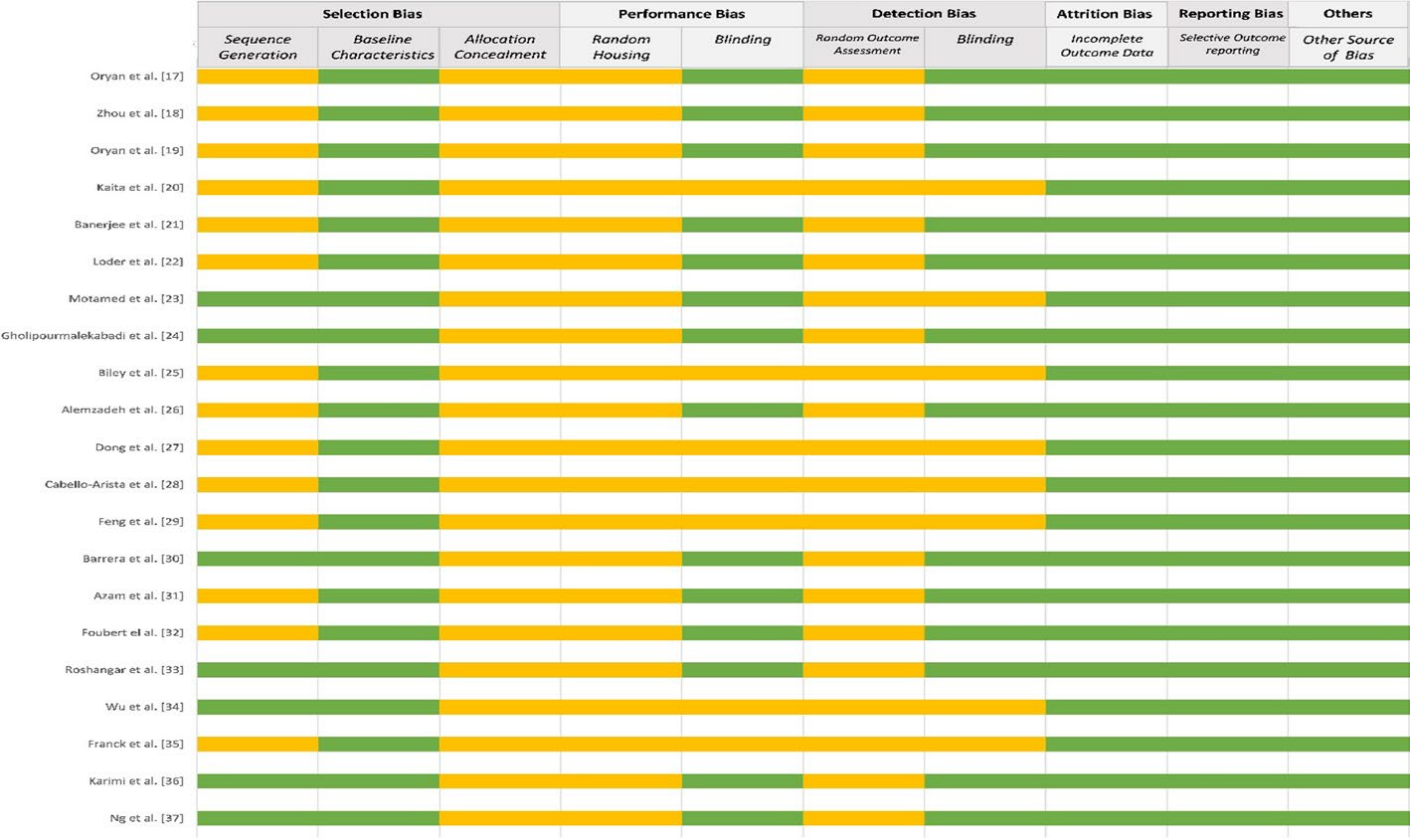
The quality of each included study according to the SYRCLE risk of bias tool for animal studies

Upon applying the SYRCLE's Risk of Bias tool to the included 21 in vivo studies, the following observations were made (Fig. 2):

In the domain of sequence generation, only seven study indicated a low risk of bias, leaving 14 studies with an unclear risk. For allocation concealment, the risk remained unclear for all included studies. All included studies showed a low risk of bias in the domain of baseline characteristics. The absence of clarity in sequence generation and allocation concealment could potentially result in selection bias.

According to the publications, all 21 studies had an unclear risk in the domain of random housing and outcome assessment. In addition, seven studies had an unclear risk of blinding bias, both in the performance and detection bias section. This could potentially affect the reliability of the results and introduce performance and detection bias.

All of the included studies demonstrated a low risk of bias in the domain of incomplete outcome data and selective outcome reporting, indicating a low risk of attrition and reporting bias.

Moreover, all of 21 included studies demonstrated a low risk of bias in the domain of other sources of bias.

In conclusion, the application of the SYRCLE's Risk of Bias tool to these 21 animal studies has provided valuable insights into the methodological strengths and weaknesses present. While the low risk of bias in areas such as baseline characteristics, incomplete outcome data, selective outcome reporting, and other sources of bias is commendable, the high level of uncertainty in key domains, notably in selection, performance, and detection bias, is a cause for concern.

## Study characteristics

### Animal models

Of 21 studies included, 10 were based on a rat model [17–19, 21, 23, 26, 29, 31, 33, 35], 10 on a mouse model [20, 22, 24, 25, 27, 28, 30, 34, 36, 37], and a pig model [32] was employed in another instance. A total of 517 animals were examined in the studies. In 13 studies, full-thickness burns were inflicted on the laboratory animals, and in the remaining eight trials, deep partial-thickness burns were induced. The injuries were established by using specific heated devices on the animals' dorsa in 17 trials. In two studies [22, 33] were the wounds created by exposure to hot liquid and in another [31] by hydrochloric acid. 12 h after the burns, P. aeruginosa infection was induced in the treatment groups in the study by Banerjee et al. [21].

### Intervention

ADSCs were injected in nine studies [17–19, 22, 25, 29, 31, 35, 36] or applied topically as wound dressings in 10 [20, 21, 23, 24, 27, 28, 32–34, 37]. In two studies, both variants were applied [26, 30]. Injections were either intradermal [17, 19, 26, 29, 35], subcutaneous [18, 22], or sub-escharal [25]. In six studies hydrogel [17, 21, 26, 27, 30, 37] was used as carriers for ADSCs. In another four the carrier was phosphate buffered saline (PBS) [18, 22, 25, 29]. Other carriers included medical honey [19], Dulbecco’s modified eagle medium (DMEM) [31, 35], human amniotic membrane [23, 24, 28], artificial dermis [20, 32], pig skin [28], and bio-printed gel scaffold [33, 34].

Please refer to Table 3 for the applied dose of ADSCs. In six studies, ADSCs were administered on the day of the burn [18, 20, 28, 29, 35, 36], in further five studies, on the day after the burn [22, 23, 25, 31, 33], in six other studies, two days [17, 19, 26, 32, 34, 37] and in one study, 9 days [21] after the burn respectively. In the study conducted by Barrera et al., the animals were treated with ADSCs five and 10 days after burning [30]. Zhou et al. compared a group that was injected with ADSCs on the day of the burn with a group in which the application was repeated on the fourth and eighth day post-burn [18]. One study did not specify the time-point of application [24].

In seven of the studies, the wounds were covered with transparent film dressings [19, 20, 27, 28, 33, 34, 37]. One study used hydrocolloid bandages [21] and another used Vaseline gauze as a secondary dressing [23]. Oryan et al. reported on the use of demineralized bone matrix to cover wounds [17]. In the study by Alemzadeh et al., acellular dermal matrix was prepared from sheep skin as a wound covering [26]. A self-adhesive absorbent dressing (Mepore) was used by Azam et al. [31]. Daily dressing with silver sulfadiazine impregnated sterile gauze was performed in the study by Karimi et al. [36]. In all other studies, no additional dressing was described or the authors did not respond to the e-mail enquiry.

### Country of study-origin

In terms of the number of studies conducted in each country, Iran [17, 19, 23, 24, 26, 33, 36] ranked highest with seven, followed by USA [21, 22, 25, 27, 30, 32] with six. China [18, 34] had two, while Taiwan [29], Japan [20], Mexico [28], Pakistan [31], Brazil [35] and Singapore [37] each had one. Accordingly, 13 studies originate from Asia [17–20, 23, 24, 26, 29, 31, 33, 34, 36, 37], seven studies from North America [21, 22, 25, 27, 28, 30, 32] and one from South America [35]. None of the studies were conducted in Europe, Africa, Oceania or Antarctica.

### Outcome

18 out of 21 studies demonstrated accelerated wound healing in the groups treated with ADSCs [17–20, 22–24, 26, 27, 29–37], with the remaining three studies reporting no differences in comparison with the control groups [21, 25, 28]. 16 studies compared the different closure rates in wounds [17–20, 23, 24, 26, 27, 29–31, 33–37], while two studies focused on the reduction in wound depth [22, 32].

13 studies have investigated the immunomodulation abilities of ADSCs during the inflammatory response [17–19, 23, 24, 26, 27, 30, 31, 33, 35–37], one of which found no difference from the control group [27]. A reduction in inflammatory cells through the use of ADSCs was demonstrated histologically in eight studies [17, 19, 23, 31, 33, 35–37]. The studies by Roshangar et al. and Karimi et al. showed a reduction in the number of cells including polymorphonuclear leukocytes and macrophages due to ADSCs application [33, 36]. In turn, in the study by Dong et al., no significant difference in macrophage or T-cell infiltration was detected in the groups treated either with or without ADSCs [27]. A decrease in pro-inflammatory cytokine interleukin 1-beta (IL-1β) after ADSCs application was shown in four studies [17, 19, 26, 31]. According to Gholipourmalekabadi et al., after administration of ADSCs, there was a significant increase in the pro-inflammatory cytokines macrophage inflammatory protein 2 (MIP-2) and tumor necrosis factor alpha 1 (TNF-α1), which returned to their physiological levels during wound healing, whereas the control group remained in the inflammatory response [24]. Further studies have confirmed the decrease of tumor necrosis factor alpha (TNF-α) [30, 31], along with other pro-inflammatory cytokines such as interleukin 6 (IL-6) [31], through the usage of ADSCs. Zhou et al. have shown an increase in the anti-inflammatory cytokine interleukin-1 receptor antagonist protein (IL-1ra) by ADSCs [18].

18 out of 21 studies involved the investigation of ADSCs in neovascularisation; 16 of these 18 demonstrated that the usage of ADSCs can support neovascularisation in vivo [17–22, 24–27, 29–34]. Only one study revealed a diminished neovascularisation due to the use of ADSCs [36], while another showed no effect [35]. For the research, tissue biopsies were harvested from within the wound areas. Evidence for the formation of the new vascular network was obtained either by haematoxylin and eosin [17, 19, 22–24, 26, 27, 32, 33] and Masson’s trichrome [18, 20, 24, 25, 27, 30, 32] staining, or by using specific antibodies including CD31 [18, 22, 24, 25, 27, 29, 30, 32, 34], CD34 [33], isolectinB4 (ILB4) [20], neural/glial antigen 2 (NG2) and von Willebrand factor [21] as well as vascular endothelial growth factor a1 (VEGFa1) and vascular endothelial growth factor receptor 2 (VEGFR2) [24]. In addition, several studies have demonstrated increased secretion of various proangiogenic growth factors including vascular endothelial growth factor (VEGF) [18, 24, 30, 31], basic fibroblast growth factor (bFGF) [17, 19, 24, 26, 31], hepatocyte growth factor (HGF) [31], hypoxia-inducible factor 1-alpha (HIF-1α) [31] and IL-1β [24] after the application of ADSCs.

15 studies have investigated the utility of ADSCs for granulation tissue formation [17–19, 21, 22, 24–26, 28, 31–36]. Enhanced granulation tissue formation in the ADSC groups compared to the control groups was demonstrated histologically in four studies [19, 21, 24, 31]. Cabello-Arista et al. reported an increase in granulation tissue in animals treated with human amnion and ADSCs, whereas the addition of ADSCs to porcine skin reduced granulation tissue formation [28]. An increase in fibroblast quantities by ADSCs was also demonstrated in four studies [17, 19, 26, 36]. Using green fluorescent protein (GFP) labelling, Zhou et al. demonstrated that ADSCs differentiate into fibroblast-like cells in vivo [18]. Improved collagen synthesis and deposition were reported in eight studies [21, 25, 26, 28, 31–35]. Immunohistochemically, Zhou et al. found a higher level of ki67-positive cells in the dermis of ADSCs-treated animals [18], whereas no difference was found between the various groups in the studies by Loder et al.[22].

The supportive role of ADSCs in re-epithelialisation has been investigated in 15 publications [17–19, 21–26, 28, 30, 31, 33, 34, 36]. Of these, 11 authors reported improved re-epithelialisation induced by ADSCs [17, 19, 21, 23, 24, 26, 30, 31, 33, 34], while the other four detected no difference compared with the control groups [18, 22, 25, 28]. The re-epithelialisation was investigated by histology [17, 19, 21, 23, 26, 28, 30, 34], immunohistochemistry [22], fluorescence microscopy [18] and comparison of 2-D photos [25, 28, 34]. An enhanced transforming growth factor beta (TGF-β) level 14 days after treatment with ADSCs, which returned to normal after 28 days, was observed in four studies [17, 19, 24, 26].

11 studies have reported on the influence of ADSCs during the remodelling phase [17, 19, 21, 24–27, 29, 30, 33, 37]. Barrera et al. reported significant smaller scars in ADSCs-treated animals compared to control groups [30]. Gholipourmalekabadi et. al found an approximate scar elevation index (SEI) in ADSCs treated wounds as in healthy skin. The authors assume that ADSCs are able to significantly reduce collagen expression and thus scar formation [24]. A significantly higher collagen type I to type III ratio in ADSCs treated animals was demonstrated in three studies [21, 27, 28]. Five studies document a more organized mature collagen in ADSCs treated groups compared with controls [17, 19, 21, 26, 33]. Furthermore, an increased collagen density by ADSCs was observed in four studies [19, 24, 26, 33], while a separate study found no difference between ADSCs and control groups [30].

**Fig. 3:**
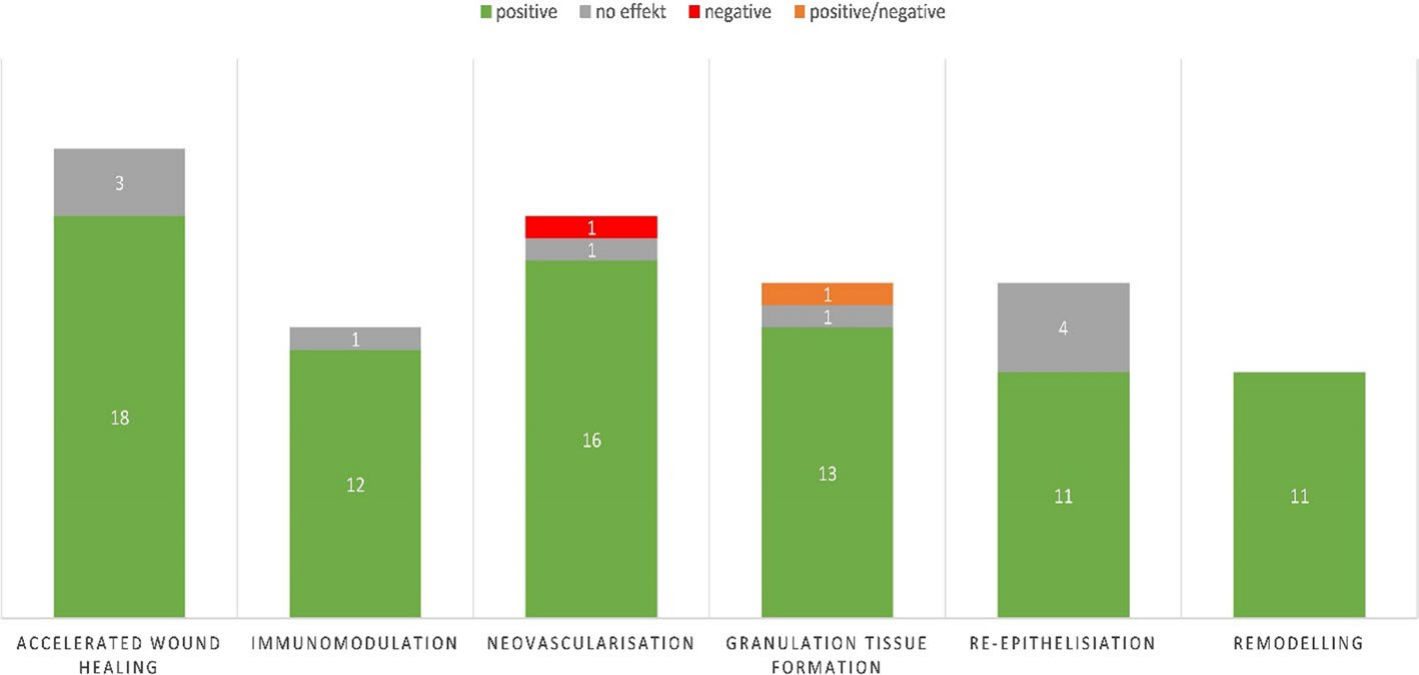
Studies examining the influence of ADSCs on wound healing and its respective phases. Green: ADSCs had a positive effect during this phase, grey: ADSCs had no effect on this phase, red: ADSCs had a negative effect during this phase, orange: The effect was positive or negative depending on the carrier substance

**Fig. 4:**
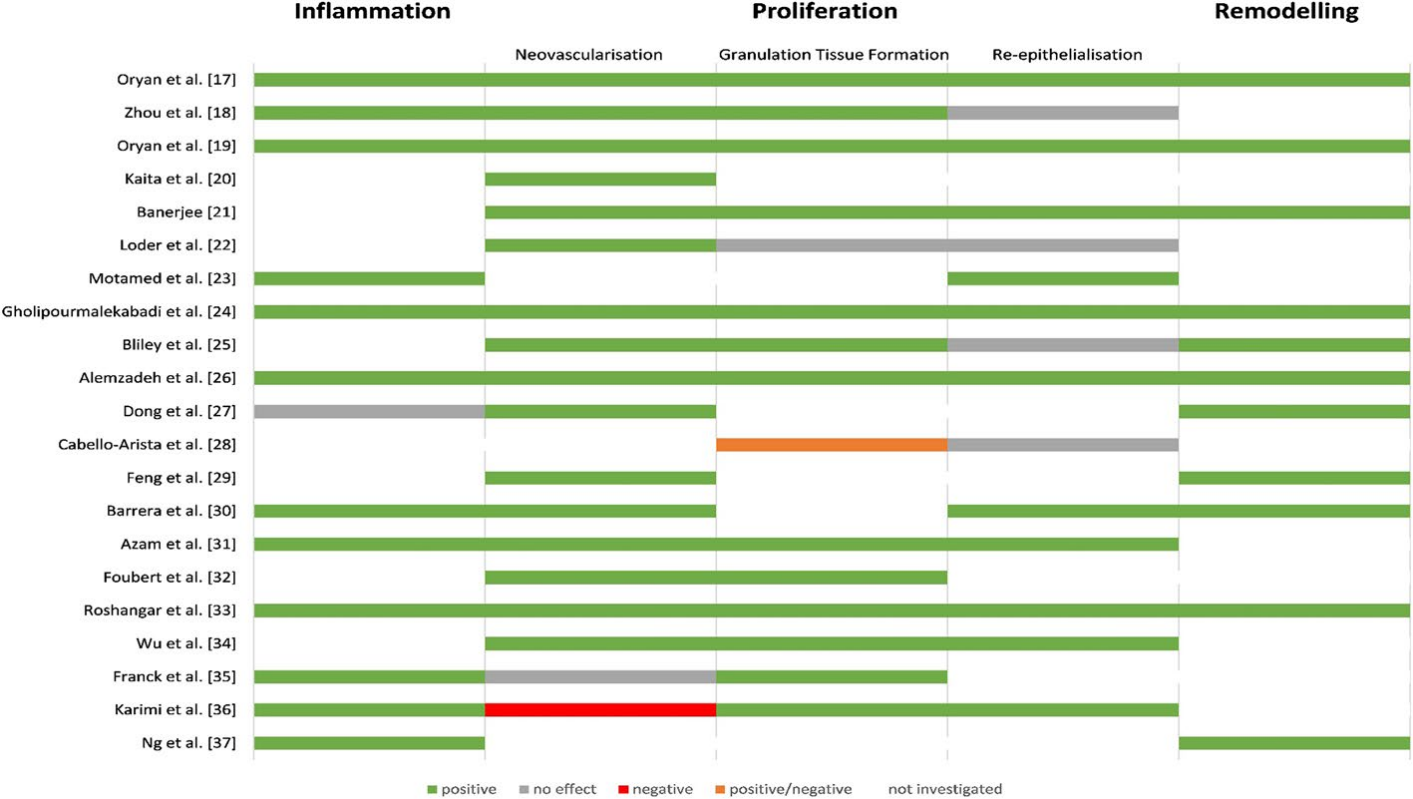
Analysis of the ADSCs associated improvement according to the wound healing phases. Green: ADSCs had a positive effect during this phase, grey: ADSCs had no effect on this phase, red: ADSCs had a negative effect during this phase, orange: The effect was positive or negative depending on the carrier substance, no colour: this phase was not investigated by the authors

Dong et al. found a significant reduction in myofibroblasts by using alpha-smooth muscle actin (α-SMA) staining [27]. No differences in α-SMA levels were observed between the ADSC and control groups in the study by Bliley and colleagues. Therefore, a significant increase in peroxisome proliferator-activated receptor gamma (PPARg) gene expression was observed in the ADSCS group at all test time points in this study [25]. In one study, elevated levels of matrix metalloproteinases 1 (MMP-1) and 2 (MMP-2) were detected [24]. Barrera et al. found that the expression of profibrotic tissue inhibitor of metalloproteinase 1 (TIMP-1) was significantly downregulated by ADSCs. Inhibition of excessive scarring by down-regulation of TGF-β1 and bFGF genes on day 28 after wounding was addressed by Alemzadeh et al. [26]. ADSC-associated hair follicle regeneration was observed in five studies [19, 24, 25, 29, 37]. The impact of ADSCs on wound healing and its respective phases is delineated in Fig. 3, while Fig. 4 is dedicated to the presentation of the findings from the included studies across these phases.

## Discussion

The result of our systematic review indicates a significant positive impact on different aspects of the wound healing process, including the initial inflammatory response, neovascularisation, granulation tissue formation, re-epithelialisation, and the remodelling phase. However, because of the remarkable variability among the studies, the possibility of conducting a meta-analysis was precluded.

The inflammatory response plays a fundamental role in wound healing and serves as the primary defence mechanism against microorganisms [38]. In severe burns, this response can be extensive and uncontrolled, leading to an augmented inflammation, which results in delayed wound healing [1, 39], and hypertrophic scar formation [1, 40–42].

The study conducted by Gholipourmalekabadi et al. demonstrated that the application of ADSCs promotes the initial inflammatory phase by stimulating the production of pro-inflammatory cytokines. This response subsequently diminishes over time, with the control group maintaining a sustained inflammatory state [24]. Based on this finding, it can be concluded that ADSCs first facilitate the immune response by promoting the inflammatory process, and then attenuate the extensive inflammatory response usually associated with severe burns to ensure a smooth transition to the proliferative phase.

Following a severe burn injury, the systemic inflammatory response encompasses the release of large quantities of pro-inflammatory cytokines such as IL-1β, MIP-2, IL-6 or TNF-α [43, 44]. Increased IL-1β delays wound healing by stimulating inflammasome activity in macrophages and inducing inflammation in other cells, hindering the polarization into the anti-inflammatory M2 phenotype [45]. MIP-2 acts as a chemokine and is secreted in response to infection or injury by cells including macrophages and monocytes. It exhibits pro-inflammatory effects by promoting the recruitment and activation of neutrophils, supporting inflammatory reactions, thus leading to tissue damage [46]. IL-6 is instrumental in triggering the acute inflammatory response. It is also essential for the transition into chronic inflammation by being the key stimulator for most acute-phase proteins, and by modifying leukocyte infiltration [47, 48]. Elevated levels of TNF-α are associated with decreased neovascularisation, cell migration and proliferation, and increased apoptosis [49]. Several of the studies included in this review, showed that the effects of ADSCs in reducing the levels of the pro-inflammatory cytokines IL-1β, MIP-2, IL-6 and TNF-α1 in animals with burn injuries [17, 19, 24, 26, 30, 31].

Furthermore, the majority of the included studies investigating the immunomodulatory capabilities of ADSCs during the inflammatory response have shown that ADSCs reduce the number of inflammatory cells [17, 19, 23, 24, 26, 27, 30, 31, 35].

Severe burn injuries with a large-scale surface area significantly heighten the risk of infection due to compromised immune response and disrupted skin barriers [1]. It would be interesting to analyse the effects of ADSCs on inflammation in the study conducted by Banerjee et al., in which the burns of experimental animals were infected with Pseudomonas aeruginosa [21]. However, the impact of ADSCs on infection-induced inflammation was not taken into account in their analysis. Instead, their focus was on examining the antimicrobial effect of chitosan microspheres loaded with silver sulfadiazine.

However, even if there are no results regarding the intentional bacterial infection of burns, one can summarily state that ADSCs appear to initially promote immunomodulation by enhancing the initial inflammatory response. Subsequently, they ensure that inflammation remains regulated, which is crucial for the transition to the proliferative phase and important for the progression of the physiological healing process, thus preventing the development of chronic wounds and pathological scars [42, 50–52].

The proliferative phase is distinguished by neovascularisation, the formation of granulation tissue, and re-epithelialisation. The majority of studies have indicated that ADSCs promote the development and formation of new blood vessels, resulting in enhanced neovascularisation. Furthermore, multiple studies have highlighted the incidence of the elevated secretion of diverse proangiogenic growth factors, such as VEGF [18, 24, 30, 31], bFGF [17, 19, 24, 26, 31], HGF and HIF-1α [31]. VEGF has a dual impact on endothelial cells, both stimulating their differentiation from endothelial progenitor cells and enhancing their migratory capacity, proliferation, and ability to organise into functional vascular tubules [12, 53–55]. HGF has the ability to induce the production of VEGF, and acts as a potent mitogen for endothelial cells, by interacting synergistically with VEGF [56, 57]. bFGF also supports the migration and proliferation of endothelial cells [58, 59]. HIF-1 activation serves as a primary stimulus for neovascularisation through blood vessel growth and remodelling, inducing important pro-angiogenic factors such as VEGF, angiopoietin 2 (Ang-2), and stromal cell-derived factor 1 (SDF-1). Furthermore, HIF-1 plays a contributory role in oxygen and nutrient delivery to hypoxic tissues, and thus enhancing cell survival [60, 61].

Neovascularisation plays a pivotal role in wound healing, by supplying oxygen and essential nutrients to developing tissues [54, 62, 63], while decreased local neovascularisation leads to impaired wound healing [63, 64]. The result of our review demonstrated that ADSCs support the neovascularisation process in burns.

Another important step in the wound healing process is the formation of granulation tissue. The creation of this new tissue is facilitated by fibroblasts which deposit extracellular matrix (ECM) components into the wound. These latter then become main components of the new granulation tissue, alongside the new blood vessels and fibroblasts themselves [38, 50, 51, 65]. The resulting newly formed tissue fills the wound gap and provides a scaffold for cell adhesion, migration, growth and differentiation during wound healing, thus enabling re-epithelialisation [50, 66, 67].

Several of the studies included in our review demonstrate that ADSCs result in an elevation of TGF-β1 levels on the 14th day after the initial injury, followed by a significant reduction by day 28 of the healing process [17, 19, 24, 26]. TGF- β1 plays a crucial role in various aspects of wound healing. It is instrumental in cellular migration, particularly for cell types like fibroblasts and keratinocytes, facilitating their movement towards the wound site. Furthermore, TGF-β1 contributes to the deposition of the ECM, which is essential for the structural support and organization of the newly formed tissue [68–70]. A multitude of included studies demonstrated enhanced granulation tissue formation [19, 21, 24, 31] and re-epithelialisation [17, 19, 21, 23, 24, 26, 30, 31, 33, 36] through the utilisation of ADSCs in burns. Interestingly, Cabello-Arista et al. revealed contrasting effects of ADSCs on granulation tissue formation depending on their carrier. The treatment with human amnion and ADSCs resulted in an increase in granulation tissue. However, when ADSCs were added to porcine skin, a reduction in granulation formation ensued [28]. These findings suggest that the interplay between ADSCs and their carrier may have varying effects depending on the material, and further research is warranted to optimize their therapeutic potential.

The application of ADSCs has been demonstrated to enhance the number of fibroblasts, according to several studies [17, 19, 26, 36]. bFGF is known to stimulate the proliferation of fibroblasts and induce the formation of granulation tissue [71, 72], and its levels have been reported to increase through the application of ADSCs [17, 19, 24, 26]. Another potential mechanism for the rise in fibroblasts is the differentiation of ADSCs into these cells, as mentioned by Zhou et al. [18]. This thesis is supported by several in vitro [10, 73–76] and other in vivo studies [74, 75]. According to Gersch et al., ADSC-differentiated fibroblasts surpass the performance of primary fibroblasts by exhibiting accelerated wound infiltration, heightened expression of ECM markers such as elastin and fibronectin, while reducing levels of scar tissue markers including α-SMA and MMP-1 [76].

One of the primary ECM components synthesized by fibroblasts is collagen, which provides structural support and strength to tissues. It plays a crucial role in wound healing by promoting tissue repair, wound closure, and eventually scar formation [38, 51, 65]. Numerous included studies have evidenced that ADSCs elicit an augmentation in collagen synthesis [21, 25, 26, 28, 34]. It is noteworthy that the accurate balancing of collagen synthesis is of paramount importance in attainment of wound healing. Insufficient collagen synthesis may inhibit wound closure and tissue repair, while excessive collagen production expedites pathological scar formation [41, 42, 77, 78]. Thus, sufficient collagen synthesis assists in the minimisation of scar formation and promotes more physiological tissue regeneration.

Furthermore, in the process of physiological healing process, a balance between deposition and degradation of the synthesised collagen is crucial [42]. MMPs play a primary role in ensuring this balance is achieved [79]. It has been observed that hypertrophic scars are associated with a decrease in the expression of MMP-1, along with elevated levels of TIMP-1 [79, 80]. The latter of which functions as an inhibitor of specific MMPs. It is noteworthy that the expression of TIMP-1 is stimulated by MMP activity [80–82]. Barrera et al. reported a decrease in TIMP-1 expression by ADSCs in burn injuries, which could have implications for hypertrophic scar formation [30]. The decreased expression of TIMP-1 by ADSCs suggests a potential mechanism by which the balance between MMPs and their inhibitors could be modulated by these cells. Through the reduction in TIMP-1 levels, a more favourable environment for MMP activity may be assisted by ADSCs. This result aligns with those reported by Gholipourmalekabadi et al., who observed elevated level of MMP-1 and MMP-2, which is associated with the degradation of various ECM components [24]. Thus its upregulation supports tissue remodelling, but can also foster extensive scar formation in the event of excessive levels. The presence of elevated MMP-2 levels in conjunction with increased MMP-1 and decreased TIMP-1 expression suggests a complex interplay between these factors and ADSCs in the regulation of scar formation after burns. These findings provide further evidence for the potential role of ADSCs in the modulation of MMP expression and their involvement in scar formation. While ADSCs may have beneficial effects on certain aspects of wound healing, further investigation is required to assess their potential influence on myofibroblasts, the expression of MMPs, and subsequent impact on scar formation.

If this interplay fails to operate effectively, an imbalance occurs, resulting in excessive or disorganised collagen deposition may result in hypertrophic or keloid scars [41, 42, 77, 83, 84], which can be aesthetically undesirable and functionally limiting. In several included studies, it was observed that ADSCs-treated groups exhibited well-organised and mature collagen bundles compared to the control groups [17, 19, 21, 26]. In physiological wound healing, the initial type III collagen is converted into mature type I collagen during the remodelling phase, resulting in strengthened wound integrity [83, 85]. Conversely, the progression of hypertrophic scars is characterized by a downregulation in collagen I expression alongside an excessive upregulation in collagen III [86]. Multiple studies included in our review consistently indicated an elevated collagen type I to type III ratio [21, 27, 28].

Moreover, hypertrophic scars are characterised by an elevated abundance of myofibroblasts, which express α-SMA and undergo apoptosis during the physiological wound healing but persist in hypertrophic scar formation [42, 84, 87, 88]. Dong et al. demonstrated a significant decrease in the population of myofibroblasts through the reduction of α-SMA [27]. Various further in vivo studies have exhibited the suppression of α-SMA levels and diminished scarring resulting from the administration of ADSCs [89–95].

Several studies have demonstrated that ADSCs treatment leads to a decrease in TGF-β1 levels concomitant with an elevation of bFGF, during remodelling [17, 19, 26]. This fact is of great interest, since TGF-β1 promotes the differentiation of fibroblasts into myofibroblasts [96, 97], while bFGF is known to inhibit extensive scar formation [98, 99].

Currently, hair follicle regeneration in full-thickness wounds continues to present a challenge in regenerative medicine [100, 101]. Whilst the body has the innate ability to repair certain tissues, such as the skin, hair follicles have a limited capacity for regeneration, especially in deep wounds involving the dermis [102]. In full-thickness burns, the destruction extends to the whole dermis [1] involving its appendages including hair follicles, the loss or damage of which can inhibit their regrowth [103–105]. Interestingly, five studies reported hair follicle regeneration in ADSCs-treated burns [19, 24, 25, 29, 37]. In four of these studies, the regeneration of hair follicles, which are usually damaged beyond repair, was observed in full-thickness burn wounds [19, 24, 25, 37]. This process namely wound-induced hair neogenesis (WIHN) is of particular interest in the field of regenerative medicine, as the restoration of hair growth in such wounds can significantly improve the aesthetic outcome and functional recovery. WIHN was first described in the middle of the twentieth century in various mammals [106–109] and was rediscovered by Ito et al. [110] in 2007, who demonstrated the development of completely new hair follicles in wounded mice. Several recent studies focusing on WIHN subsequently emerged [111–117]. According to several studies, full-thickness wounds with a diameter of at least 1 cm lead to neogenesis of hair follicles, while smaller full-thickness wounds heal with a hairless and adipose-free scar [110–114]. This largely aligns with our research, as, in three of the included studies, the full-thickness wound diameter was at least 1 cm [19, 24, 25]. Due to contraction in rodent wound healing, the edges of the hair-bearing areas are frequently distorted, giving the simulation of pre-existing hair follicles being encircled by scar tissue, thereby creating a false impression of WIHN [102]. Therefore, a detailed examination is of utmost importance to determine whether it is indeed WIHN. Recent insights suggest that adipocytes and their precursors are involved in hair follicle regeneration [118]. However, this insight necessitates comprehensive research, and further studies are imperative to understand the role of adipocyte lineage cells in hair follicle regeneration.

Despite their favorable properties in wound healing, ADSCs are presently used in burn care for experimental purposes only. Autologous skin grafting is still considered the gold standard for the treatment of severe burns [4]. Several studies have demonstrated that ADSCs support the therapeutic efficacy of split-thickness skin grafts in the treatment of burns [119–121]. Both Gao et al., and Foubert et al., have found that ADSCs can significantly enhance the elasticity of the split-thickness skin grafts, resulting in an improvement of skin texture and functionality [120, 121]. According to the research conducted by Osamu et al., the application of ADSCs significantly enhances skin graft take and inhibits transplant shrinkage throughout the healing process [119]. In addition, the studies indicate that ADSCs foster skin neovascularisation, enhance skin thickness, and expedite wound epithelialisation [119–121].

In summary, ADSCs are a promising candidate for future therapeutic approaches in the treatment of burns. All of these experiments demonstrated aspects of ADSCs that positively influence the inflammatory response, cell proliferation and migration, neovascularisation, granulation tissue formation and re-epithelialisation, as well as remodelling. However, the validity of all these results must be critically scrutinized, since most of the included studies are conducted in mice and rats. Rodent wound models are often considered limited because of the perception that rodents have a loose skin and heal primarily by contraction, offering a fast wound closure, while humans heal by re-epithelialisation [50, 122]. Nevertheless, rodents are the most extensively investigated animals in the field of burn research, primarily due to their ease of handling, rapid reproduction, and standardisation options, offering the significant benefit of accelerated healing process, which enhances research efficiency and reduces mortality [123, 124]. Rodent burn models are particularly suited for local phenomena investigations such as wound inflammation and application of various dressings [123]. Furthermore, rodents offer the opportunity to investigate the cellular architecture and interaction on wound healing, acknowledging differences from human biology [125, 126]. Whilst Chen et al. argue that re-epithelialisation in rodents is measurable [127], the predominant approach for examining re-epithelialisation involves manipulation through splinting, which minimises contraction to emulate human wound healing [122, 123, 125]. However, with the exception of two articles [25, 27], in which the use of splinting was negated, none of the included studies in our review reporting on its usage. Consequently, the effect of ADSCs on the re-epithelisation process remains unclear. Additionally, the reliability of comparing rodents to humans in the research of hair follicle regeneration remains questionable due to the significant differences in dermal cell biology [117, 128]. However, the investigation of ADSCs in the treatment of burns is at a very early experimental stage and the mechanism of their action is currently not completely understood. Further studies in species with skin structures and healing physiology similar to humans, such as pigs [122, 129], are essential to determine the efficiency of ADSCs in burn wound healing. It is crucial to comprehend the precise processes involved in the interplay between ADSCs and the different phases in wound healing in order to develop targeted therapeutic strategies for optimizing burn care. Additional research is needed to elucidate the specific signalling pathways and cellular interactions influenced by ADSCs in the context of wound healing and scar formation.

### Limitations

Our systematic review has some inherent limitations. For one, only articles written in English language were taken in account within this review. As a result, some papers that are not available in English have not been considered. Only items discovered through our search strategy retrieved from PubMed, Web of Science and Embase, or manual search in relevant journals were considered, with the possibility of missed publications. Another addition to the limitations is that our review is limited to articles published before 30th September 2022. Since science is a dynamic process leading to constant developments, papers published after this date have not been considered within our review. in spite of literature screening by two investigators, a possible wrongful exclusion cannot be ruled out. A major limitation is that most of the studies were conducted on rodents, which makes reproducibility and transfer in a clinical context challenging. Finally, despite the usage of the SYRCLE's Risk of Bias tool and independent assessment by two reviewers, it's important to acknowledge that bias assessment can be inherently subjective, and so the results should be interpreted with this in mind.

## Conclusion

In conclusion, it appears that adipose-derived stem cells demonstrate remarkable efficacy in the field of regenerative medicine, offering positive support throughout wound healing. However, the usage of ADSCs in the treatment of burns is still in the early experimental stage and the majority of the studies were conducted in rodents. The included studies have revealed varied approaches when considering cell count, administration protocol, and carrier selection. Given the foundational insights, it is imperative to elucidate the optimal administration protocol for ADSCs and to discern the most appropriate carrier, considering the specific state of the wound. Hence, further investigations are necessary to investigate the efficacy of ADSCs in the treatments of burns and its potential adoption in clinical settings.

## Data Availability

For data requests please contact the corresponding author.

## Abbreviations

MSCs: Mesenchymal stem cells
ADSCs: Adipose-derived stem cells
PRISMA: Preferred Reporting Items for Systematic Reviews and Meta-Analyses
PBS: Phosphate buffered saline
DMEM: Dulbecco’s modified eagle medium
IL-1β: Interleukin 1-beta
MIP-2: Macrophage inflammatory protein 2
TNF-α1: Tumor necrosis factor alpha 1
TNF-α: Tumor necrosis factor alpha
IL-6: Interleukin 6
IL-1ra: Interleukin-1 receptor antagonist protein
ILB4: Isolectin B4
NG2: Neural/glial antigen 2
VEGFa1: Vascular endothelial growth factor a1
VEGFR2: Vascular endothelial growth factor receptor 2
VEGF: Vascular endothelial growth factor
bFGF: Basic fibroblast growth factor
HGF: Hepatocyte growth factor
HIF-1α: Hypoxia-inducible factor 1-alpha
GFP: Green fluorescent protein
TGF-β: Transforming growth factor beta
SEI: Scar elevation index
α-SMA: Alpha-smooth muscle actin
PPARg: Peroxisome proliferator-activated receptor gamma
MMP-1: Matrix metalloproteinases 1
MMP-2: Matrix metalloproteinases 2
TIMP-1: Profibrotic tissue inhibitor of metalloproteinase 1
ANG-2: Angiopoietin 2
SDF-1: Stromal cell-derived factor 1
ECM: Extracellular matrix
WIHN: Wound-induced hair neogenesis
